# Binaphthyl-Based Macrocycles as Optical Sensors for Aromatic Diphenols

**DOI:** 10.3390/molecules25030514

**Published:** 2020-01-24

**Authors:** Stefano Piacentini, Marco Caricato, Aurora Pacini, Andrea Nitti, Dario Pasini

**Affiliations:** Department of Chemistry and INSTM Research Unit, University of Pavia, Via Taramelli 12, 27100 Pavia, Italy; stefano.piacentini01@universitadipavia.it (S.P.); gcarica@libero.it (M.C.); au.pacini@gmail.com (A.P.); andrea.nitti01@universitadipavia.it (A.N.)

**Keywords:** chirality, macrocycles, binaphthyl, circular dichroism, chiroptical sensors

## Abstract

The synthesis of several rigid, homochiral organic macrocycles possessing, respectively, average molecular *D*_2_ and *D*_3_ symmetries, is described. They have been obtained from aromatic dicarboxylic acids, in combination with an axially-chiral, suitable dibenzylic alcohol, derived from 1,1′-binaphthyl-2,2′-diol (BINOL) using one-pot esterification reactions in good isolated yields. NMR and circular dichroism (CD) spectroscopies detect the structural and shape variability in the scaffolds, reflected both in terms of the changes in chemical shifts and the shape of selected proton resonances, and in terms of the variation of the CD signature related to the dihedral angle defined by the binaphthyl units embedded in the rigid cyclic skeleton. The *D*_2_ cyclic adducts are able to form stable complexes with aromatic diphenols, with binding strengths that are dependent on small variations in the spacing units, and therefore on the shapes of the internal cavities of the cyclic structures.

## 1. Introduction

Large, shape-persistent macrocyclic structures are of increasing interest for applications in the field of supramolecular chemistry, molecular recognition, and nanoscience [1,2,3,4]. Shape persistency relates to the conformational stability and rigidity of the covalent cyclic structure. Those features are fundamental properties to be sought in order to enhance the recognition/complexation properties toward suitable inclusion guests. This issue is also stringent because macrocycles can self-assemble in stable organic nanotubes by supramolecular organization in the third dimension [5,6,7].

Chirality has been exploited in combination with shape-persistent macrocyclic structures both for sensing and recognition, and for the assembly of chiral nanotubes [8]. Chiroptical sensors are a very appealing and emerging class of sensors, in which the appearance or the modulation of the circular dichroism (CD) signal, respectively, represents the chiroptical readout [9,10,11]. CD spectroscopy can offer better levels of detection when compared with optical spectroscopies and electrochemistry-based methods, and it is frequently used in biosensing, where high sensitivities are required [12,13]. Fluorescent sensors, however, have been shown to be able to afford excellent sensitivity levels and can realize enantioselective recognition [14,15,16,17]. The most frequent chiroptical sensing mechanisms are either the interaction between a nonracemic chiral substrate and a chromophoric probe, which is silent with respect to circular dichroism (CD) spectroscopy, or the interaction between an achiral analyte and a chromophoric CD-active probe [18,19,20]. Atropoisomerically-chiral compounds are particularly suitable for application in chiroptical sensing, since the expression of chirality is embedded into a chromophore, resulting in peculiar CD activity [21].

We have previously reported, in a sequence of papers, our approach to imparting chirality to covalent cyclic structures for chiroptical sensing and the assembly of chiral nanotubes, based on the use of 1,1′-binaphthyl-2,2′-diol (BINOL)-based molecular synthons [22,23,24,25,26,27,28,29]. One of our key concepts was the use of BINOL-based probes exhibiting a “spring-like” behavior, with an intense CD signal modulation upon subtle changes in their conformation upon binding, which was successful in the chiroptical detection of species as diverse as anions, cations, or C_60_. In this paper, we report on the synthesis of novel macrocycles, in which the phenolic functionalities of the binaphthyl units are protected by *t*-butyloxycarbonyl groups, and on their unexpected binding of π-electron rich aromatic diphenols.

## 2. Results and Discussion

### 2.1. Design, Synthesis, and NMR Characterization

**Scheme 1:**
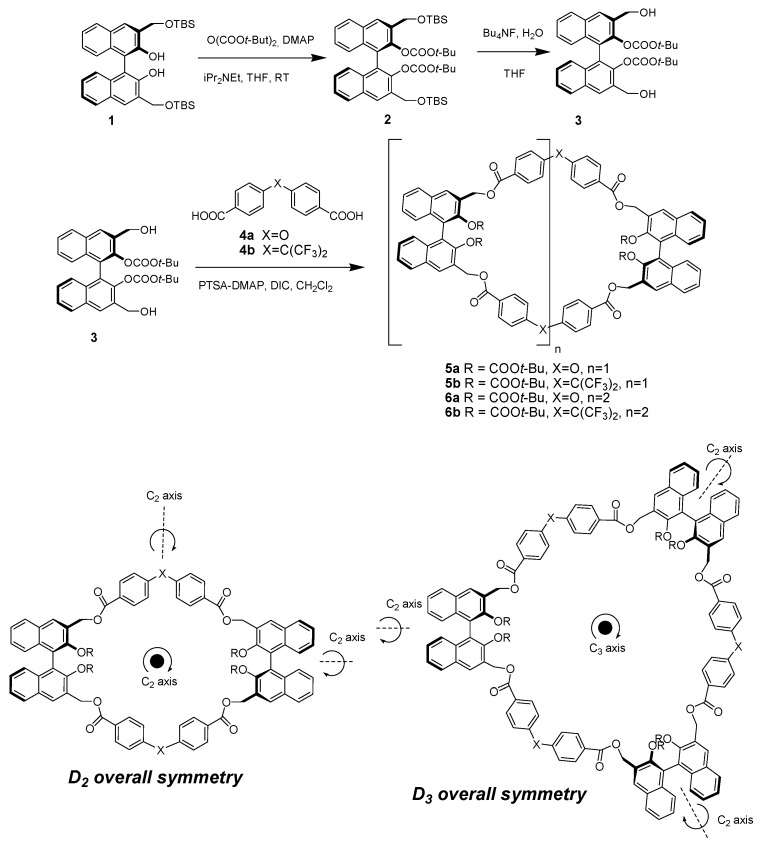
Synthesis of suitable precursors and cyclization to form macrocycles **5** and **6**.

In our molecular design, and in line with the literature and our own previous work, a modification of the binaphthyl synthons followed the elaboration of the 2,2′ and 3,3′ positions, amongst the most reactive and easy to functionalize on the naphthyl skeletons, in order to insert suitable functionalities for both cyclization and molecular recognition. The use of high-yielding, well established synthetic protocols for the rapid covalent construction of the cyclic moieties was also an unavoidable premise. The synthesis of the key precursors and of the macrocycle is shown in Scheme 1.

In our design strategy, macrocycles **5** and **6** could be precursors, via the unmasking of the phenolic functionalities of very interesting supramolecular receptors with acidic hydroxyl groups pointing directly inside the chiral cavities. We have previously described the synthesis of resolved, optically pure BINOL derivatives bearing dimethyl-*t*butylsilyl(TBS)-protected benzylic diols in the 3,3′-positions of the binaphthyl moieties (compound (*R*)-**1**) [30]. Since direct cyclization in the presence of unmasked phenols by means of esterification had already been performed in our group without success [26], protection of the 2,2′ phenolic positions was needed. It was achieved in high yields by means of *t*-butyloxycarbonyl protecting groups, which could be cleaved after cyclization in mild acidic conditions. The selective deprotection of the external TBS protecting groups afforded key compound (*R*)-**3** in good yields. A previously published esterification protocol was used for the cyclization reaction through the direct formation of ester bonds from the difunctional diols and dicarboxylic acids. We utilized commercially available dicarboxylic acid spacers **4a** and **4b**, in which the aryl ether bridge and the quaternary carbon atom between two aromatic rings, respectively, impose a defined dihedral angle, and therefore define internal, noncollapsible macrocyclic cavities. The cyclization of elaborated precursor (*R*)-**3** afforded, when using **4a**, the homochiral macrocycles (*RR*)-**5a** and (*RRR*)-**6a**, incorporating, respectively, 2 and 3 binaphthyl homochiral units, respectively, and possessing overall *D*_2_ and *D*_3_ molecular symmetry, in 25% and 10% yields. In the case of spacer **4b**, the yields for (*RR*)-**5b** and (*RRR*)-**6b** were very similar: 26% and 10%. The macrocycles were obtained in pure form after purification by column chromatography, with the larger macrocycles eluting last, and unequivocally characterized by NMR spectroscopy and mass spectrometry (vide infra). The products were isolated as white waxy solids, which could be stored at room temperature for several months with no sign of decomposition. Unmasking of the phenolic functionalities by deprotection of (*RR*)-**5a** was attempted using various classical literature methods (TFA in CH_2_Cl_2_, HCl in THF or dioxane) [31,32] but failed to give clean products and proved poorly reproducible. It is likely that the acid-labile benzylic esters were cleaved by the acids, causing the formation of large amount of byproducts.

**Figure 1:**
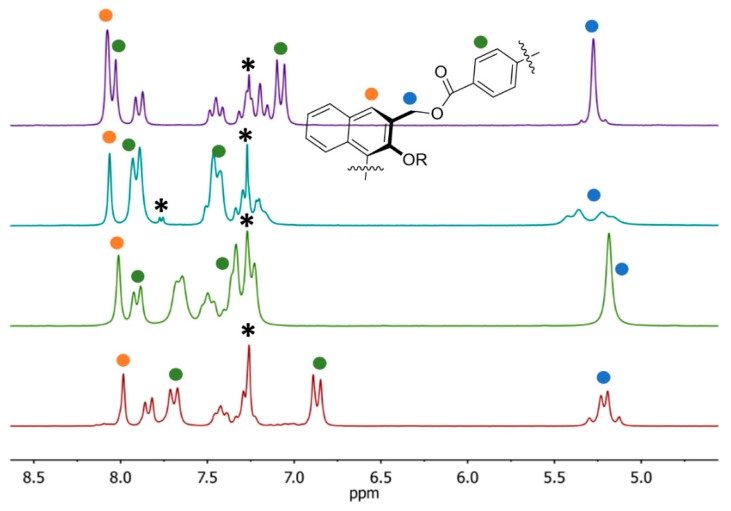
Aromatic and CH_2_ benzylic ester region for the ^1^H NMR spectra (CDCl_3_, 200 MHz, 25 °C) of macrocycles (from top to bottom) **5a**, **5b**, **6b**, **6a**. The asterisk marks impurities and residual solvent.

The room temperature ^1^H NMR spectra for all homochiral cyclic compounds showed the expected simple patterns and the presence of only one set of signals for each group of symmetry-related proton resonances, revealing that all possible dynamic processes (slow rotation around the aryl–aryl bond or conformational locking of the aromatic ester residues) were, as expected, fast on the NMR timescale at this temperature. The comparison between the spectra is reported in Figure 1, and the full NMR characterization is reported in the Supplementary Materials section.

It was interesting, however, to detect differences in the CH_2_ benzylic proton resonances, which are diastereotopic, and they appeared either as a collapsed AB system, in the case of the aryl ether spaced macrocycles **5a** and **6b**, or as a defined AB system, in the case of the quaternary carbon spaced macrocycles **5b** and **6a**. The two diastereotopic methylene protons of each macrocycle define an angle for the H–C–H bond imparted by the quaternary carbon atom. However, the flexibility allowed by the overall backbone of the different macrocycles can make those protons experience substantially different environments (i.e., one of them can be more or less buried into the cavity and experience more or less pronounced shielding effects by the aromatic portions of the spacing units). The differences can therefore be a direct consequence of the internal geometry of the cavities, defined either by the aryl ether spacing units or the fluorine-containing spacing units between the binaphthyl moieties.

### 2.2. Spectroscopic and Complexation Studies

**Figure 2:**
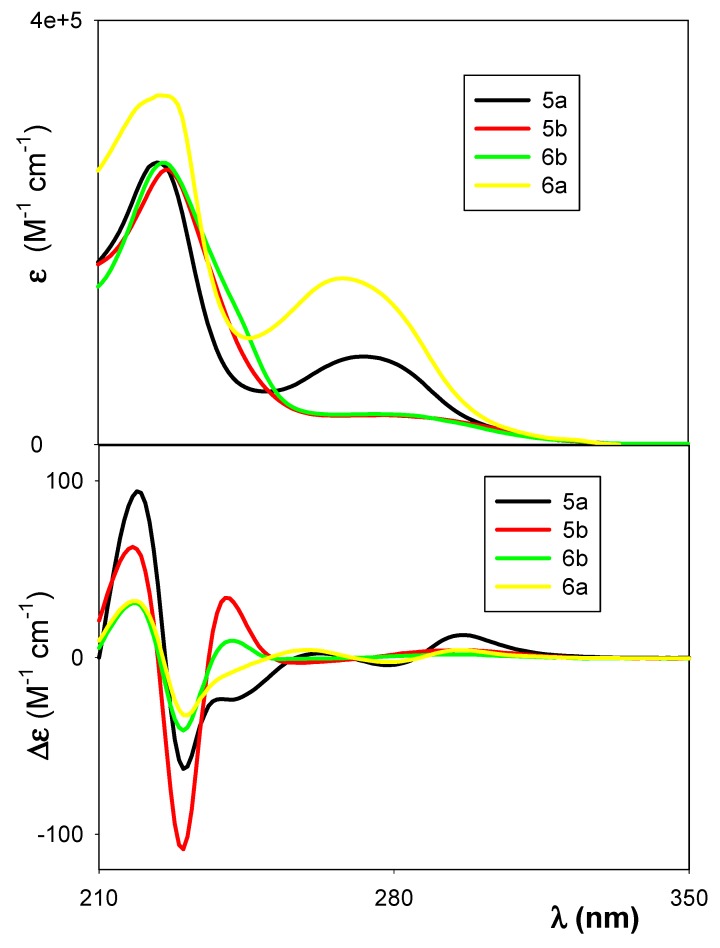
UV (**top**) and circular dichroism (CD) (**bottom**) spectra of macrocycles **5** and **6**. Concentrations: 10^−5^ M for UV and 2.2–2.6 10^−6^ M for CD spectroscopy in MeCN.

The UV/Vis absorption spectra of the macrocyclic compounds (Figure 2) described in this paper show a band centered around 230 nm, typical of the binaphthyl chromophore, with molar absorptivity values within the range of those already reported for this class of chromophores (ca. 10^5^ M^−1^ cm^−1^ per binaphthyl unit in MeCN).

The other low energy band is associated with the chromophore belonging to the spacing units of **4a** and **4b**. Circular dichroism (CD) spectroscopy of macrocycles **5** and **6** showed the exciton couplet typical of binaphthyl moieties, corresponding to the maximum absorption band in the UV/Vis spectra (ca. 230 nm for all compounds). The intensity of the low energy component of the couplet (at 230–240 nm) ranged between values of −40 for **6b** and −100 for **5b**; for O-substituted 2,2′-binaphthol derivatives, these values have been directly related to variations of the dihedral angle between the naphthyl units as a consequence of the steric hindrance of the substituents in the 2,2′-positions [33]. Since compounds **5** and **6** possessed the same substituent (O*t*Bu) in the 2,2′-positions, the differences between the above mentioned values should be ascribed to variations of the average dihedral angle of the binaphthyl units as a consequence of their incorporation in cyclic structures of differing sizes and structural flexibility, or, in other words, as a consequence of a more or less intense buttressing effect of the neighboring 3,3′ benzylic ester positions. The binaphthyl units acted therefore as CD reporters for the strain of the internal cavities of the macrocycles. In fact, the smaller, more strained macrocycles **5a** and **5b** showed more induced CD activity in the chromophoric region of the spacing units, whereas the larger, more flexible macrocycles **6a** and **6b** did not show such activity, or only in a marginal way.

Our failure to deprotect the phenolic functionalities did not allow us to explore the supramolecular chemistry of the phenol-based macrocycles [34], but the carbonyl rich framework of the macrocycles, and their large cavities, prompted us to explore, nevertheless, their supramolecular properties. Titration of C_60_ of any of the synthesized macrocyclic hosts resulted in no binding being detected by UV/Vis spectroscopy, following protocols used by us in previous publications [26].

Hydroxyl groups have not been exhaustively targeted as supramolecular handles in the design of noncovalent receptors, so that literature precedents are not abundant. The only notable exception is perhaps in the field of receptors for monosaccharides [35]. We reasoned that phenols could be suitable substrates to examine for recognition, since the aromatic portion of the molecule could be included in the cavity of the macrocycle and the hydroxyl functionality could bind through hydrogen bonding to one of the oxygen groups (ether or carbonyl) exposed within the macrocyclic framework.

**Figure 3:**
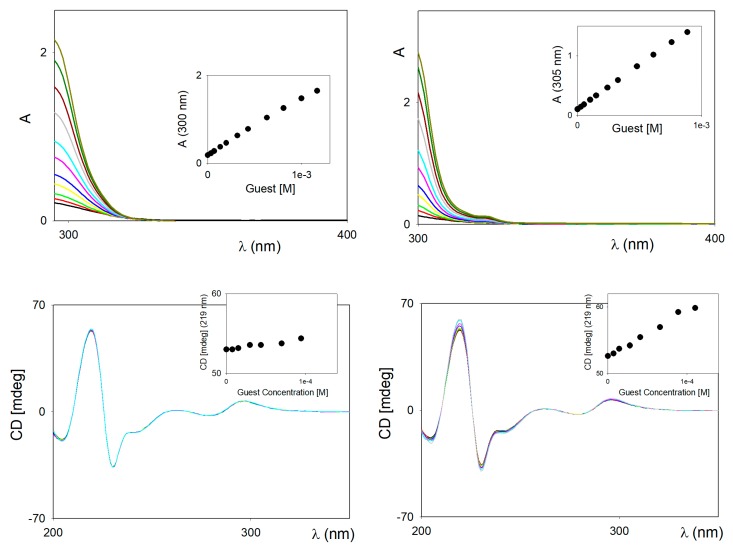
UV (**top**) and CD (**bottom**) titration of macrocycle **5a** and hydroquinone (**left**) and 1,4-dimethoxybenzene (**right**). Concentrations: 10^−5^ M for UV and 10^−6^ M for CD spectroscopy in MeCN.

By titrating a solution of macrocycle **5a** in MeCN (5 × 10^−5^ M) with increasing amounts of hydroquinone (1,4-dihydroxybenzene), a variation of the structure of the absorption band could be detected (Figure 3). Self-aggregation of the guest could be excluded, since variable concentration UV/Vis spectra were essentially superimposable, and the absorption of the guest at the wavelength used for the binding isotherm was negligible. The binding isotherm could be fitted with high confidence to a 1:1 binding model. When the titration was carried out using 1,4-dimethoxybenzene, a considerably smaller value for the 1:1 binding constant was obtained (Table 1, entries 1 and 2).

Titrations using **5a** monitored by CD spectroscopy (at a slightly lower concentration) afforded no variation in the case of hydroquinone as the guest (Figure 3). Instead, in the case of 1,4-dimethoxybenzene, a detectable variation upon binding of the exciton couplet band attributable to the π-extended binaphthyl units, centered at 230 nm, was observed. The variation could be related to a modification of the dihedral angle between the naphthyl planes of the binaphthyl units, the presence of a strong supramolecular interaction of the guest molecule with the binaphthyl units, a conformational rearrangement of the macrocycle upon binding to the guest, or even a combination of some of these factors. Although the dihedral angle variation and the conformational rearrangement must have been marginal, the detectable change in the exciton couple once again reinforces the “spring-like” concept for such axially-chiral probes and forecasts great utility in sensing. In fact, CD spectroscopy of the guest alone did not show any signal, be it achiral or other. We also investigated the binding properties of the other aromatic diphenol hydroquinone isomers and obtained a binding constant of the same order of magnitude (Table 1). With the differently spaced macrocycle **5b**, binding constants of the same magnitude as that of **5a** were recorded, with 1:1 binding observed with high confidence outputs.

It is likely that two supramolecular interactions (i.e., solvophobic interactions and hydrogen bonding interactions in the case of the diphenols) were both playing a role in the formation of the host-guest complexes and not always positively cooperating. The fact that the CD response occurred only in the presence of the most sterically hindered dimethoxy substrate suggests that the tight inclusion of the aromatic portion of the guest substrate into the suitably sized receptor was necessary for a steric interaction with the binaphthyl units, a change of their conformation, and a derived change of the CD output.

## 3. Materials and Methods

### 3.1. General Experimental Details

All commercially available compounds were purchased from commercial sources and used as received. Aromatic phenols and methyl ethers were recrystallized before use. Compounds PTSA-DMAP [36] and (*R*)-**1** [28] were prepared according to literature procedures [37]. THF (Na) and CH_2_Cl_2_ (CaH_2_) were dried before use. Analytical thin layer chromatography was performed on silica gel, using chromophore-loaded, commercially available plates. Flash chromatography was carried out using silica gel (pore size 60 Å, 230–400 mesh). ^1^H and ^13^C NMR spectra were recorded from solutions in CDCl_3_ on a 200 or 300 MHz spectrometer, with the solvent residual proton signal or tetramethylsilane as a standard. The UV/Vis spectroscopic studies were recorded using commercially available spectrophotometers. Mass spectra were recorded using an Agilent Technologies electrospray ionization (ESI-MS) instrument. Optical rotations were measured on a polarimeter with a sodium lamp (*λ* = 589 nm) and are reported as follows: [α]^rt^_D_ (*c* = g (100 mL solvent) ^−1^). CD spectroscopy was performed at 25 °C at a scanning speed of 50 nm·min^−1^, and the spectra were background corrected.

### 3.2. General Procedure for the UV, CD and NMR Titration Experiments

The titration experiments (UV/Vis, Circular Dichroism (CD), and ^1^H-NMR) were conducted as follows. To a stock solution of the host molecule (solution A) in pure CH_3_CN (UV/Vis spectroscopic grade or HPLC grade; CD_3_CN in the case of ^1^H-NMR) were added several aliquots of the guest compound (solution B). Solution B was formed by the guest molecule at a higher concentration dissolved in solution A, in order to maintain the ligand always at the same, constant concentration.

In the case of a 1:1 binding isotherm, by employing a nonlinear fitting curve program, the plot of the absorbance against the metal concentration x was fitted by Equation (1), thus affording the value of the association constant k_a_.

(1) $$A=\left(\varepsilon_{c}\;-\;\varepsilon_{s}\right)\frac{k_{a}\left(C+x\right)+1\;-{\left[{\left[k_{a}\left(C+x\right)+1\right]}^{2}\;-\;4{k_{a}}^{2}\mathrm{Cx}\right]}^{0.5}}{2k_{a}}+\;\varepsilon_{s}C$$

where A is the measured absorbance, x is the total concentration of titrant added, ε_c_ is the molar absorptivity of the complex, ε_s_ is the molar absorbivity of the substrate at the desired wavelength (which could be directly determined), C is the total concentration of the titrate (which is a constant quantity, usually the macrocycle), and *k*_a_ is the association constant for the 1:1 complex.

**Compound***(R)*-**2**. (*R*)-**1** (709 mg, 1.23 mmol, 1 equiv.) was added to a solution of DMAP (catalytic amount) and THF dry (10 mL). After 10 min, the *i*Pr_2_NEt (95 mg, 0.740 mmol, 0.6 equiv.) and di-BOC anhydride (860 mg, 3.95 mmol, 3.2 equiv.) were added to the mixture. After 15 h of stirring at R.T. and a TLC (hexane/ethyl acetate 98:2), the reaction mixture was concentrated in vacuo, and the crude product was purified by column chromatography (hexane/ethyl acetate 98:2) to yield compound **2** (907 mg, 95%). [α]^rt^_D_ = −69° (*c* = 0.002, CH_2_Cl_2_). ^1^H-NMR (300 MHz, CDCl_3_) *δ* = 8.15 (m, 2 H; CH binaphthyl), 7.90 (m, 2 H; CH binaphthyl), 7.42 (m, 4 H; *J* = 6 Hz, CH binaphthyl), 7.24 (t, 2 H; *J* = 6 Hz, CH binaphthyl), 5.00 (d, 2 H; *J =* 15 Hz, –CH_2_O–), 4.89 (d, 2 H; *J =* 15 Hz, –CH_2_O–), 1.04 (s, 18 H; –OCOOC(CH_3_)_3_), 1.01 (s, 18 H; –SiMe_2_C(CH_3_)_3_), 0.18 (s, 6 H; –Si(CH_3_)_2_tBu). ^13^C-NMR (75 MHz, CDCl_3_) *δ* = 150.3 (C=O), 144.5 (Cq), 133.2 (Cq), 132.1 (Cq), 131.7 (Cq), 127.6 (CH), 126.7 (CH), 126.3 (CH), 125.7 (CH), 125.5 (CH), 123.7 (Cq), 82.7 (Cq), 77.1 (Cq), 60.2 (CH_2_), 26.8 (CH_3_), 25.9 (CH_3_), −5.4 (CH_3_). MS(ESI): *m*/*z* = 797 [*M* + Na]^+^ (100%). Anal. calcd. for C_44_H_62_O_8_Si_2_: C 68.2%, H 8.1%; found C 68.3%, H 8.0%.

**Compound***(R)*-**3**. Bu_4_NF solution 1M in hexane (10 mL, 10 mmol, 9 equiv.) was added to a solution of compound **2** (907 mg, 1.17 mmol, 1 equiv.), THF (20 mL), and H_2_O (2 mL), and stirred overnight at R.T. After checking by TLC (hexane/ethyl acetate 9:1) the full conversion of compound **2**, the reaction mixture was quenched with water (20 mL), and THF was removed in vacuo, extracted with Et_2_O (3 × 30 mL), and dried over Na_2_SO_4_. The solution was filtered and concentrated in vacuo and the crude product was purified by column chromatography (hexane/ethyl acetate 9:1) to yield compound **3** (571 mg, 89%). [α]^rt^_D_ = +12° (*c* = 0.003, CH_2_Cl_2_). ^1^H-NMR (300 MHz, CDCl_3_) *δ* = 8.06 (m, 2 H; CH binaphthyl), 7.90 (m, 2 H; CH binaphthyl), 7.39 (t, 4 H, *J* = 6 Hz, CH binaphthyl), 7.33 (t, 2 H; *J* = 6 Hz, CH binaphthyl), 7.11 (d, 2 H; *J* = 3 Hz, CH binaphthyl), 5.43 (m, 4 H; –CH_2_O–), 1.54 (s, 18 H; OCOOC(CH_3_)_3_). ^13^C-NMR (75 MHz, CDCl_3_) *δ* = 153.7 (C=O), 150.7 (Cq), 133.3 (Cq), 130.9 (CH), 128.9 (Cq), 128.4 (CH), 127.5 (CH), 124.3 (Cq), 124.1 (CH), 124.1 (CH), 111.9 (Cq), 82.6 (Cq), 77.1 (Cq), 64.5 (CH_2_), 27.7 (CH_3_). MS(ESI): *m*/*z* 1114.5 [2 *M* + Na]^+^ (65%), 569 [*M* + Na]^+^ (35%). Anal. calcd. for C_32_ H_34_O_8_: C 70.3%, H 6.3%; found C 70.3%, H 6.0%

**Macrocycles** (*RR*)-**5a** and (*RRR*)-**6a.** Compound **3** (289 mg, 0.529 mmol, 1 equiv.) and PTSA-DMAP (328 mg, 1.06 mmol, 2 equiv.) were mixed in 15 mL of CH_2_Cl_2_ under N_2_. Then, a solution of diacid **4a** (137 mg, 0.529 mmol, 1 equiv.) in 15 mL of CH_2_Cl_2_ was added to the above solution. After 15 min DIC (245 µL, 1.59 mmol, 3 equiv.) was added. The solution was stirred under N_2_ overnight (16 h). After checking by TLC (hexane/ethyl acetate 8:2) the full conversion of compound **2**, the reaction mixture was quenched with water (30 mL), extracted with CH_2_Cl_2_ (3 × 50 mL), and dried over Na_2_SO_4_. The solution was filtered and concentrated in vacuo, and the crude product was purified by column chromatography (hexane/CH_2_Cl_2_ 2:8 for **5a** and CH_2_Cl_2_ for **6a**) to yield macrocycle **5a** (110 mg, 27%) and **6a** (40 mg, 10%). For **5a**: [α]^25^_D_ = +79° (*c* = 0.001, CH_2_Cl_2_). ^1^H NMR (CDCl_3_, 300 MHz, 25 °C) *δ* = 8.08 (s, 4 H, binaphthyl), 8.05 (d, *J* = 8.4 Hz, 4 H, phenyl), 7.89 (d, *J* = 7.5 Hz, 4 H, binaphthyl), 7.45 (t, *J* = 7.5 Hz, 4 H, binaphthyl), 7.27 (t, *J* = 7.5 Hz, 4 H, binaphthyl), 7.18 (d, *J* = 7.5 Hz, 4 H, binaphthyl), 7.08 (d, *J* = 8.5 Hz, 4 H, phenyl), 5.35–5.20 (m, 8H, Bin–CH_2_O–), 1.32 (s, 36 H, *t*-But). ^13^C NMR (CDCl_3_, 75 MHz, 25 °C) *δ =* 163.7 (Cq), 161.0 (Cq), 153.4 (Cq), 144.9 (Cq), 133.4 (Cq), 132.2 (2CH), 131.7 (Cq), 130.6 (CH), 128.3 (CH), 127.5 (CH + Cq), 126.7 (CH), 126.4 (CH), 125.1 (Cq), 124.3 (Cq), 119.2 (2CH), 82.5 (Cq *t*-But), 64.9 (CH_2_), 27.7 (3CH_3_). MS(ESI): *m*/*z* 1560.5 ([*M* + Na]^+^, 100%). Anal. calcd. for C_92_H_80_O_22_: C 71.9%, H 5.2%; found C 71.6%, H 5.0%. For **6a**: [α]^25^_D_ = +45° (*c* = 0.001, CH_2_Cl_2_). ^1^H NMR (CDCl_3_, 300 MHz, 25 °C) *δ* = 8.01 (s, 4 H; binaphthyl), 7.90 (d, *J* = 8.3 Hz, 4 H; binaphthyl), 7.65 (s, 4 H, binaphthyl), 7.49 (t, *J* = 7.4 Hz, 4 H, binaphthyl), 7.41–7.29 (m, 12 H, binaphthyl and phenyl), 7.25 (d, *J* = 10.6 Hz, 8 H, phenyl), 5.18 (s, 8 H, Bin–CH_2_O–), 1.35 (s, 36 H; *t*-Bu). ^13^C NMR (CDCl_3_, 75 MHz, 25 °C) *δ =* 167.3 (Cq), 160.4 (Cq), 152.8 (Cq), 144.3 (Cq), 132.8 (Cq), 131.6 (2CH), 131.1 (Cq), 130.0 (CH), 127.7 (CH), 126.9 (CH + Cq), 126.1 (CH), 125.8 (CH), 124.5 (Cq), 123.7 (Cq), 118.6 (2CH), 81.9 (Cq *t*-Bu), 64.3 (CH_2_), 27.1 (3CH_3_). MS(ESI): *m*/*z* 2336.3 ([*M* + Na]^+^, 100%). Anal. calcd. for C_138_H_120_O_33_: C 71.9%, H 5.2%; found C 71.6%, H 5.0%.

**Macrocycles** (*RR*)-**5b** and (*RRR*)-**6b.** They were prepared with the same procedure of **5a** and **6a**, but with compound **4b** instead of **4a**. The crude product was purified by the column chromatography to yield (*RR*)-**5b** (26%) and (*RRR*)-**6b** (10%). [α]^25^_D_ = +79° (*c* = 0.001, CH_2_Cl_2_). ^1^H NMR (CDCl_3_, 300 MHz, 25 °C) *δ* = 8.12 (s, 8 H; Py), 7.92 (m, 16 H; phenyl and binaphthyl), 7.47 (t, 4 H; binaphthyl), 7.35 (t, 4 H; binaphthyl), 7.27 (t, 4 H; binaphthyl), 6.96 (d, 8 H; phenyl), 6.51 (s br, 8 H; Py), 5.55 (q, 8 H; Bin–CH_2_O–), 4.56 (dd, 8 H; Py–CH_2_O–). ^13^C NMR (CDCl_3_, 75 MHz, 25 °C) *δ =* 165.5 (Cq), 160.2 (Cq), 154.5 (Cq), 149.0 (Cq), 145.5 (Cq), 134.2 (Cq), 132.5 (CH), 131.6 (2CH), 130.4 (Cq), 129.0 (Cq), 128.4 (CH), 127.5 (CH), 125.6 (CH), 125.4 (CH), 125.1 (Cq), 118.7 (2CH), 73.8 (CH_2_), 63.3 (CH_2_). MS(ESI): *m*/*z* 1864.5 ([*M* + Na]^+^, 100%). Anal. calcd. for C_98_H_80_F_12_O_20_: C 65.2%, H 4.5%; found C 65.6%, H 4.8%. For **6b**: [α]^25^_D_ = +39° (*c* = 0.001, CH_2_Cl_2_). ^1^H NMR (CDCl_3_, 300 MHz, 25 °C) *δ* = 8.11 (s, 4 H; binaphthyl), 8.09 (d, 4 H; binaphthyl), 7.91 (d, 8H; phenyl), 7.47 (t, 4 H; binaphthyl), 7.29 (q, 4 H; binaphthyl), 7.20 (d, 4 H; binaphthyl), 6.91 (d, 8 H; phenyl), 5.17 (s, 8 H; Bin–CH_2_O–), 1.34 (s, 36 H; *t*-But). ^13^C NMR (CDCl_3_, 75 MHz, 25 °C) *δ* *=* 167.3 (Cq), 160.4 (Cq), 152.8 (Cq), 144.3 (Cq), 132.8 (Cq), 131.6 (2CH), 131.1 (Cq), 130.0 (CH), 127.7 (CH), 126.9 (CH + Cq), 126.1 (CH), 125.8 (CH), 124.5 (Cq), 123.7 (Cq), 118.6 (2CH), 81.9 (Cq *t*-But), 64.3 (CH_2_), 27.1 (3CH_3_). MS(ESI): *m*/*z* 2796.3 ([*M* + Na]^+^, 100%).

## 4. Conclusions

We have reported the synthesis of homochiral organic macrocycles, starting from elaborated optically-active binaphthyl-based synthons and suitable, commercially available dicarboxylic acids. The syntheses are relatively straightforward and proceed affording cyclized products in high yields, also considering that no template is used. The isolated cyclic adducts comprise two or three binaphthyl units, affording molecular entities possessing overall *D*_2_ and *D*_3_ molecular symmetries, respectively. The larger macrocycles reach 3000 Da as molecular mass. We have demonstrated that NMR and CD spectroscopies are essential in order to detect the structural and shape variability in the molecular scaffolds. The *D*_2_ cyclic adducts are able to form stable complexes with aromatic diphenols, with binding strengths that are dependent on small variations in the spacing units, and therefore on the shapes of the internal cavities of the cyclic structures. In the case of methyl-protected hydroquinone, a weak chiroptical readout of the titration is possible, reinforcing the usefulness of the introduction of atropoisomerically-chiral CD reporting moieties in supramolecular receptors.

## Figures and Tables

**Table 1:** Association constant K_ass_ (M^−1^) for the 1:1 complexes between phenol-containing aromatics and macrocycles **5** measured by UV/Vis titration at 298 K in MeCN^1^.

Guest	5a	5b
1,4-dihydroxybenzene	100	140
1,4-dimethoxybenzene	38	245
1,2-dihydroxybenzene	50	380
1,3-dihydroxybenzene	180	100

^1^ Errors for the interpolation assuming a 1:1 model were <5% in all cases. See the Experimental Section for details.

## Supplementary Materials

The following are available online. Copies of NMR spectra for all new macrocycles.
