# Supplementary material for: Binaphthyl-Based Macrocycles as Optical Sensors for Aromatic Diphenols
Source: Molecules. 2020 Jan 24;25(3):514. doi: 10.3390/molecules25030514 (PMC7038072; doi:10.3390/molecules25030514)

Supplementary Materials for

Binaphthyl-Based Macrocycles as Optical Sensors for Aromatic Diphenols

Stefano Piacentini, Marco Caricato, Aurora Pacini, Andrea Nitti [1] and Dario Pasini *

Department of Chemistry and INSTM Research Unit, University of Pavia, Via Taramelli 12, 27100, Pavia, Italy;

* Correspondence: dario.pasini@unipv.it (D.P.)

Compound **5a**

^{1}HNMR (300 MHz, $CDCl_3$)

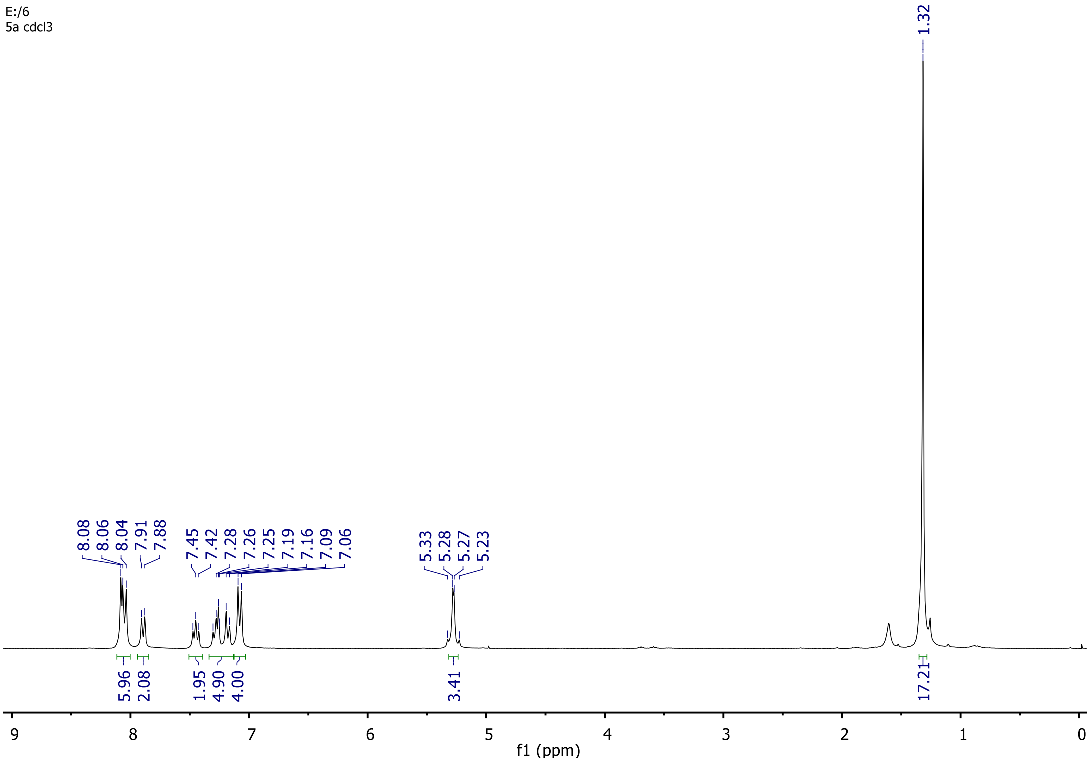

^{13}CNMR (75 MHz, $CDCl_3$)

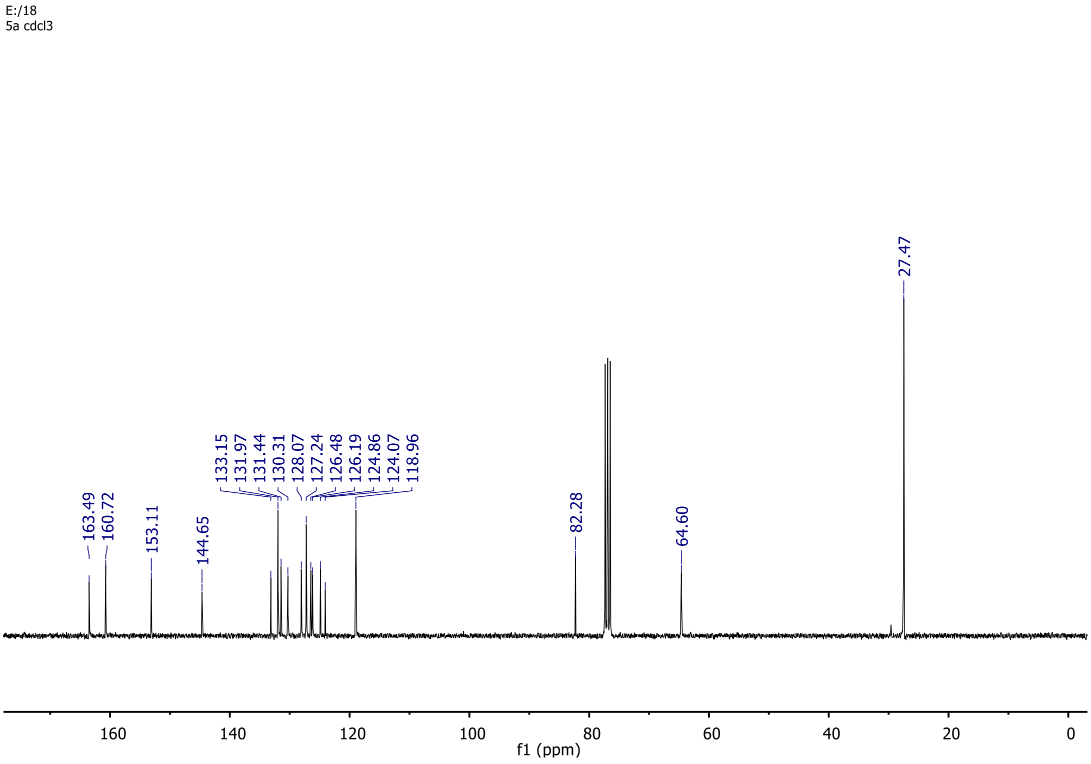

Compound **5b**

^{1}HNMR (300 MHz, $CDCl_3$)

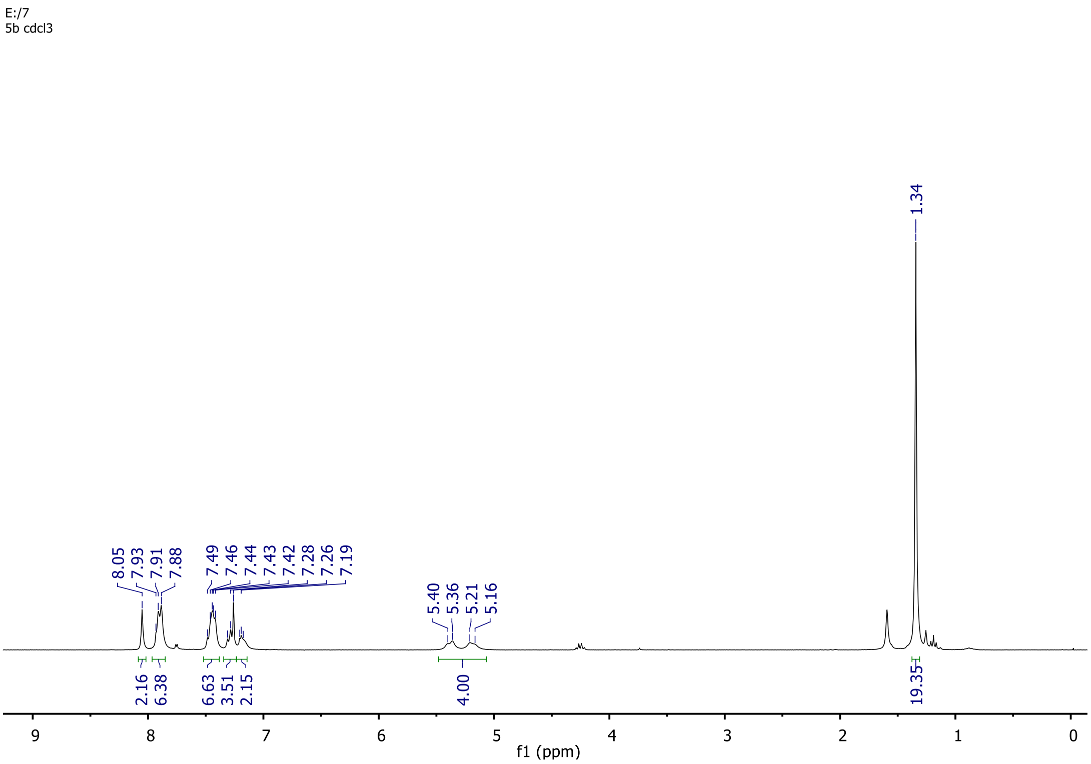

^{13}CNMR (75 MHz, $CDCl_3$)

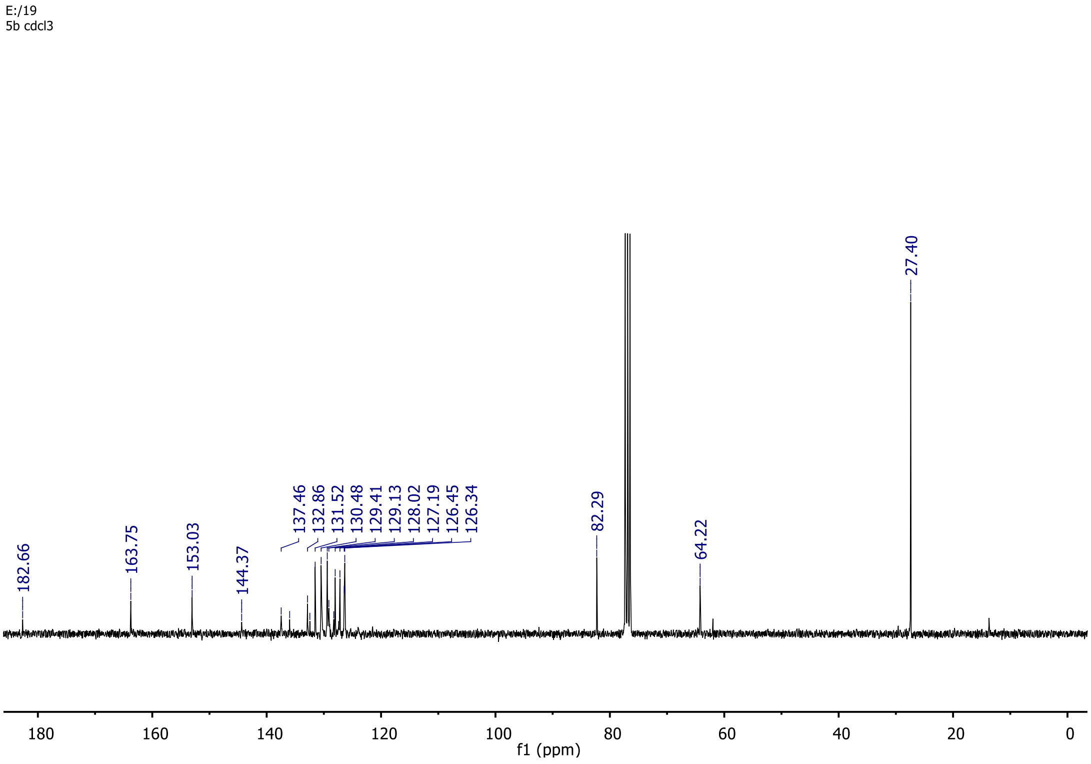

Compound **6a**

^{1}HNMR (300 MHz, $CDCl_3$)

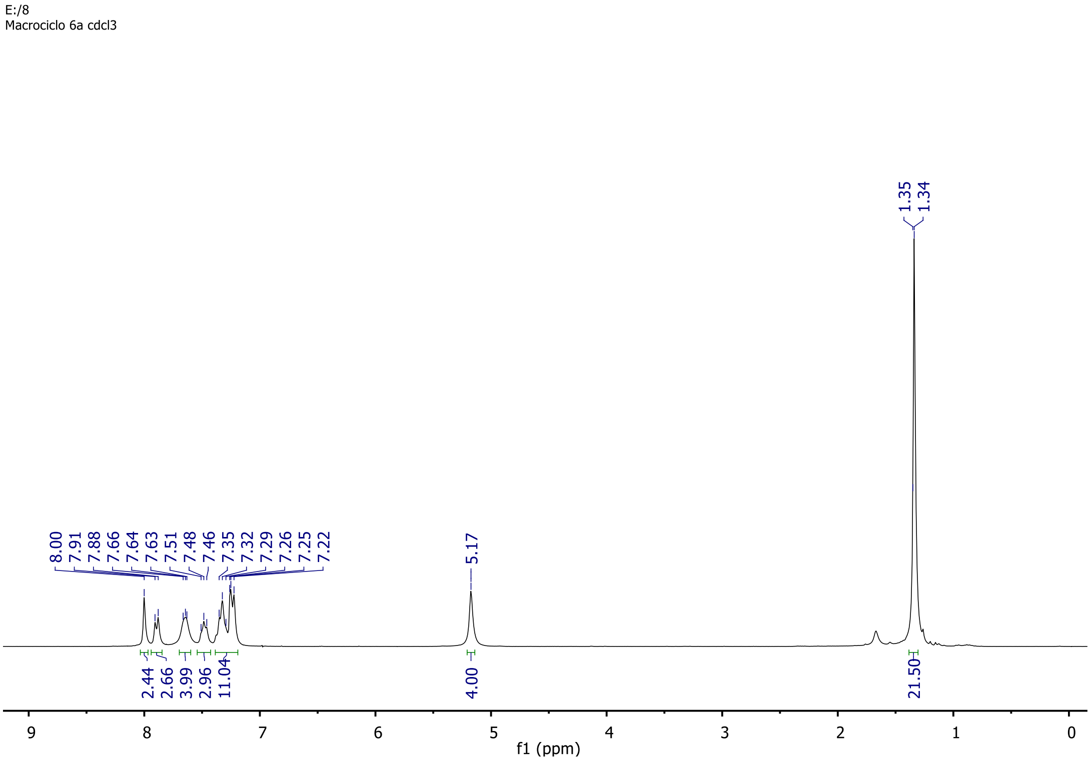

^{13}CNMR (75 MHz, $CDCl_3$)

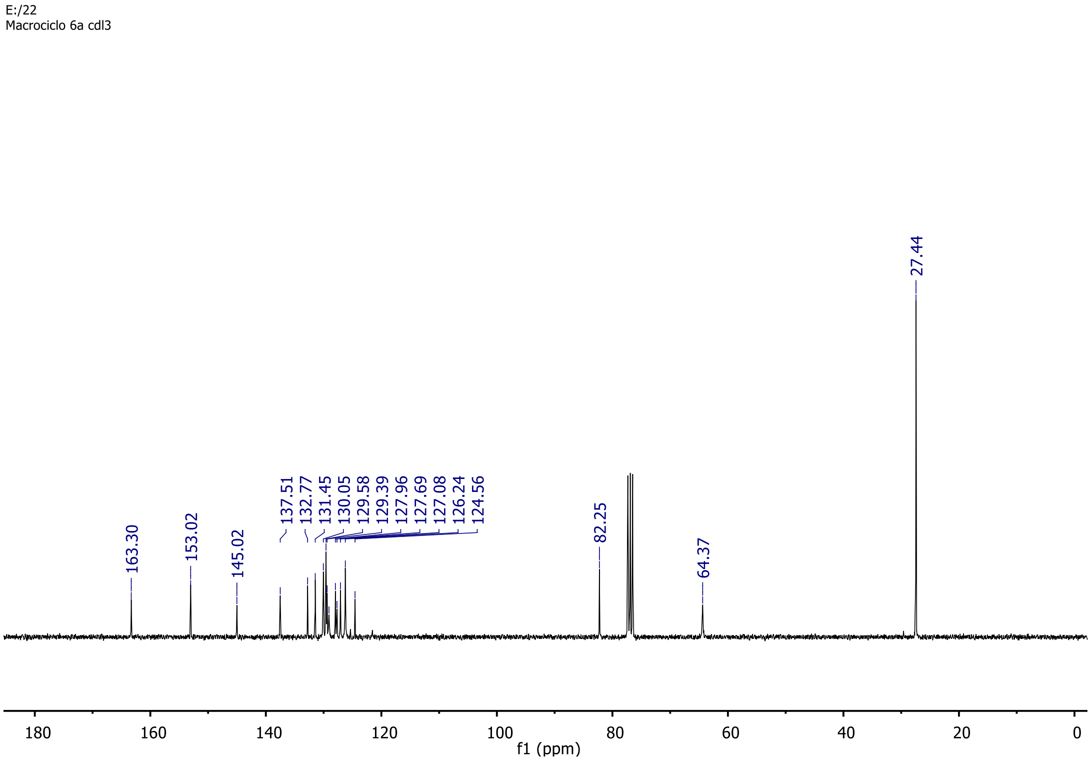

Compound **6b**

^{1}HNMR (300 MHz, $CDCl_3$)

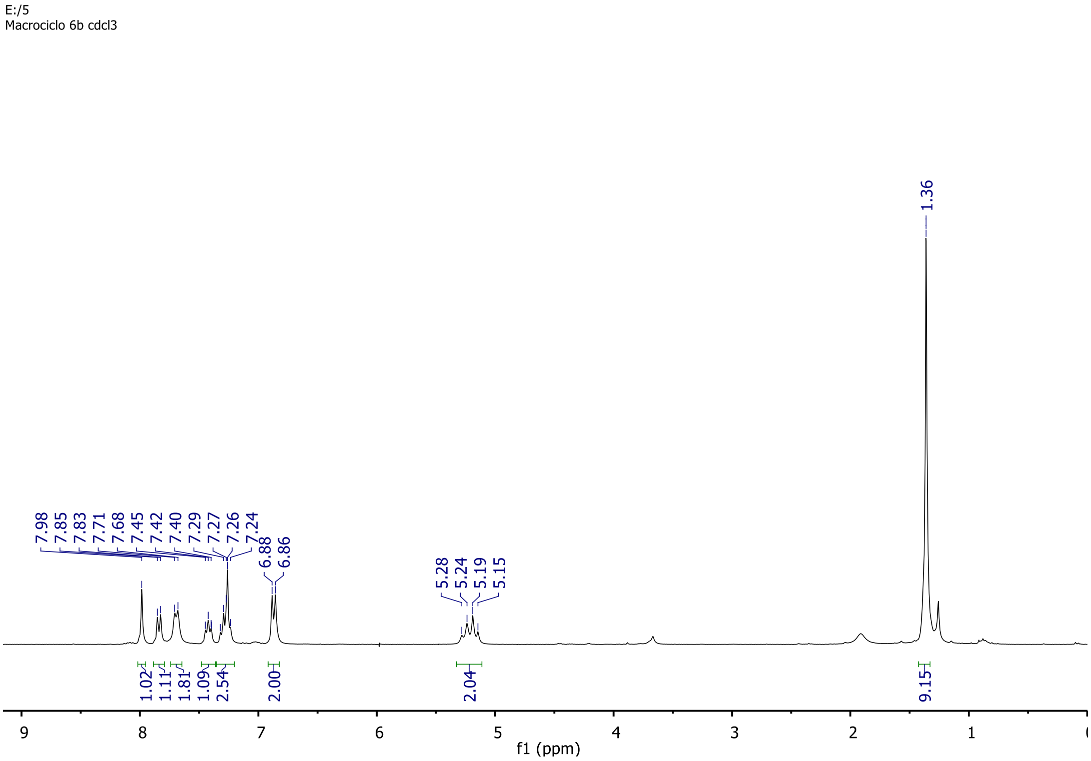

^{13}CNMR (75 MHz, $CDCl_3$)

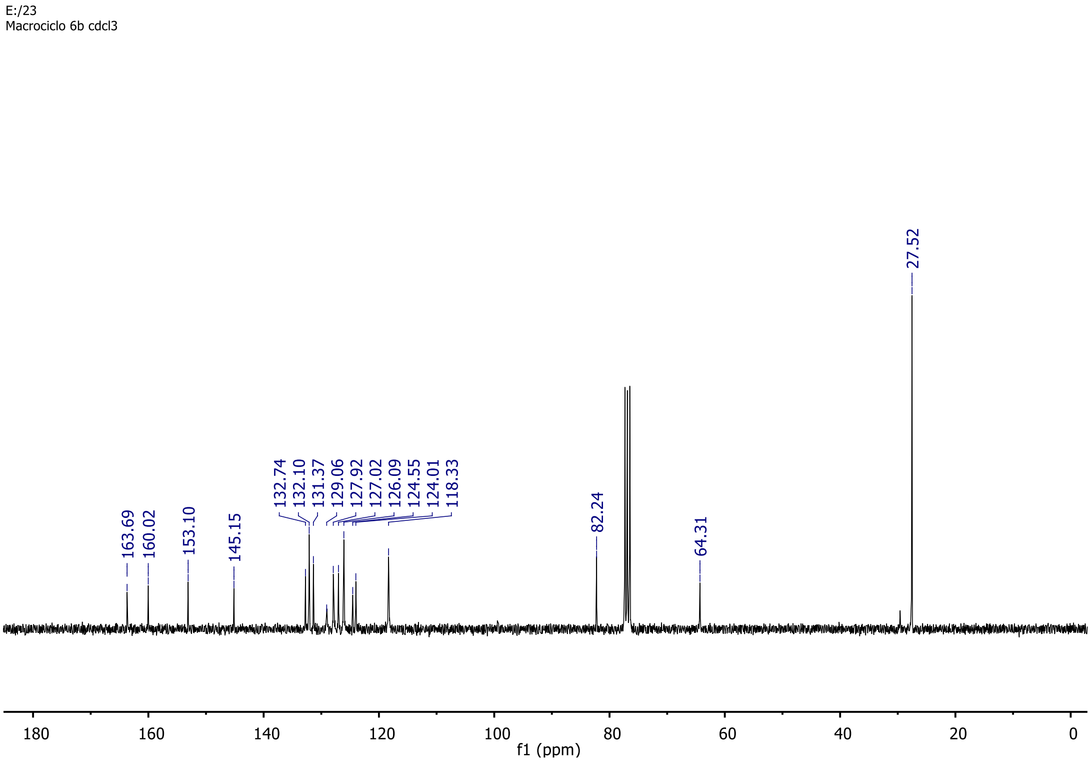

Supplement: Supplementary file 1 [file molecules-25-00514-s001.pdf]
